# Systematic review: Symptoms of parental depression and anxiety and offspring overweight

**DOI:** 10.11606/s1518-8787.2020054001731

**Published:** 2020-05-15

**Authors:** Paula Lobo Marco, Inaê Dutra Valério, Christian Loret de Mola Zanatti, Helen Gonçalves

**Affiliations:** I Universidade Federal de Pelotas Faculdade de Medicina Departamento de Medicina Social PelotasRS Brasil Universidade Federal de Pelotas. Faculdade de Medicina. Departamento de Medicina Social. Pelotas, RS, Brasil; II Universidade Federal do Rio Grande Faculdade de Medicina Departamento de Medicina Social PelotasRS Brasil Universidade Federal do Rio Grande. Faculdade de Medicina. Programa de Pós-graduação em Saúde Pública. Departamento de Medicina Social. Pelotas, RS, Brasil

**Keywords:** Mother-Child Relations, Mood Disorders, Anxiety Disorders, Pediatric Obesity, Systematic Review

## Abstract

**OBJECTIVE:**

To evaluate the existing literature on the association between parents’ depression and anxiety and their influence on their children’s weight during childhood, identifying possible mechanisms involved in this association.

**METHODS:**

A systematic search of the literature was conducted in the PubMed, PsycINFO and SciELO databases, using the following descriptors: (maternal OR mother* OR parent* OR paternal OR father) AND (“common mental disorder” OR “mental health” OR “mental disorder” OR “depressive disorder” OR depress* OR anxiety OR “anxiety disorder”) AND (child* OR pediatric OR offspring) AND (overweight OR obes* OR “body mass index” OR BMI). A total of 1,187 articles were found after peer selection.

**RESULTS:**

In total, 16 articles that met the inclusion criteria were selected for the review. Most of them investigated depressive symptoms and only three, symptoms of maternal anxiety. The evaluated studies suggested a positive association between symptoms of maternal depression and higher risk of childhood obesity. The results diverged according to the chronicity of depressive symptoms (episodic or recurrent depression) and income of the investigated country (high or middle income). Mechanisms were identified passing by quality of parenthood, affecting behaviors related to physical activity and child-feeding, as mediators of the association.

**CONCLUSIONS:**

We conclude there is evidence of a positive relationship between the occurrence of maternal symptoms of depression and anxiety and childhood obesity. It is emphasized the need for a better understanding on the effect of depressive symptoms and the contextual factors involved in this relationship so that effective intervention strategies can be implemented.

## INTRODUCTION

In the last four decades, the number of obese children and adolescents has increased tenfold in the world, from 11 million in 1975 to 124 million in 2016^1^. According to this trend, by 2022 obesity will overcome malnutrition in these phases of life^1^.

In addition to the increase in obesity in the population of children and adolescents, another contemporary global phenomenon is the high prevalence of depression and anxiety disorders noted among young people and adults^2^. Around 4.4% of the world’s population is depressed and 3.6% with anxiety disorders, reaching rates of 5.9% and 7.7%, respectively, in the Region of the Americas^2^. Between 2005 and 2015, a worldwide increase of 18% in depression and 15% in anxiety disorders occurred. Despite the differences between countries, in all of them a higher prevalence of both disorders among women is present^3^.

Obesity is a disease with multifactorial etiology^4^, including biological factors such as genetics; environmental factors, such as exposure to obesogenic environments; and psychosocial factors involving feeding, such quality of parenthood^5,6^. Regarding feed, parenthood is manifested by practices like child food control and food use as a reward, being associated with childhood obesity^6,7^.

One factor that directly influences the quality of parenthood and, as a consequence, children’s health, is the presence of mental disorders in parents^8^. Mothers with depression, generally, do not provide adequate care to their children, affecting their level of responsiveness to the child^7^. This is reflected in less healthy eating habits and less control over television viewing time and other sedentary behaviors, with low stimulation of recreational activities that require direct maternal involvement^9,10^.

Some studies have shown a relationship between mothers’ mental disorders and their children’s nutritional status^10^; in this sense, some research projects indicate that depressed women in the postnatal period tend to breastfeed for less time than recommended^13^ and to have malnourished or obese children^8^. Due to the increasing rates of depressive and anxiety disorders, it becomes of great relevance the investigation of their consequences in parent-child relations, especially during childhood, period when the child’s habits are formed.

This systematic review of the literature aimed to evaluate studies on the association between parental anxiety and depression disorders and their influence on their children’s overweight during childhood, identifying possible mechanisms involved in this association.

## METHODS

### Study Design

A systematic review of the literature was conducted, aiming to identify original articles that evaluate the association between parental depression and anxiety disorders, and overweight of children aged between 1 and 12 years.

### Methods of Search and Studies Selection

The search was conducted between November 2018 and February 2019, in the databases PubMed, SciELO and PsycINFO. The following terms were used for search: (maternal OR mother* OR parent* OR paternal OR father) AND (“common mental disorder” OR “mental health” OR “mental disorder” OR “depressive disorder” OR depress* OR anxiety OR “anxiety disorder”) AND (child* OR pediatric OR offspring) AND (overweight OR obes* OR “body mass index” OR BMI). The only restriction was that the studies were conducted in humans. Furthermore, an active search was performed in the references of the selected articles.

The review process was carried out by two independent reviewers (PLM and IDV), whose disagreements were discussed until the consensus on the inclusion of the article to compose the review.

### Inclusion Criteria

To be included in this review, the articles had to evaluate both exposure to common mental disorders (any type of depressive and/or anxiety disorders) of parents, and the outcome overweight or obesity of children, defined in the studies by accurate methods of nutritional assessment.

Notably, although depression in pregnancy and postpartum (defined for the purposes of this review as the period between childbirth and at 12 months of age) is not this study focus, studies with these exposures were included when, analyzing longitudinally, the information went further this period.

### Exclusion Criteria

Studies that did not present validated depression and anxiety measures, performed exclusively in the prenatal, gestational or within one year after childbirth, as well as those evaluating only children in the first year of life, adolescents, children with health problems that could affect weight or exclusively obese children were excluded.

### Quality Assessment

The articles in this review were evaluated for the methodological adequacy used in the sample selection, collection and analysis of data according to the Downs & Black instrument^14^ adapted for observational studies, with a scale ranging between 0 and 15 points. The following main aspects of each study were analyzed: definition of objectives and outcome measures; definition of inclusion and exclusion criteria, period and location of the study, sampling procedures (target population and description of the final sample steps), description and/or loss report, variability measures (error/standard deviation and confidence interval), description of outcome measurement methods (personnel training, calibration of appropriate instruments and clothing), and statistical analysis (appropriate statistics, control for confounding factors, and power calculation).

## RESULTS

### Selection of Studies

A total of 1,196 articles were found, adding the three databases consulted, with a total of 1,187 after deleting duplicates (n = 9). The search strategy identified 1,127 publications in the PubMed database, 48 in the PsycINFO database and 21 in the SciELO database. The selected ones were imported to an EndNote® library and, after reading the titles, 94 records were chosen by consensus among reviewers. The main reason for exclusion was the non-satisfaction of the inclusion criteria related to the outcome or exposure. Based on the abstracts reading, articles were selected for full reading. At this stage, 60 articles were excluded for not evaluating the outcome of interest, leaving 34 for complete reading. After full reading, 19 articles were excluded for the following reasons: evaluation of exposure outside the period of interest (n = 11), population composed only of obese children or at risk of obesity (n = 2), older than required (n = 2), maternal mental disorders analyzed as a moderating variable (n = 1) and not using a cut-off point to classify the nutritional status of children (n = 3). A study was included after active search in the references of the selected articles, adding 16 articles for inclusion in this literature review.

**Figure 1:**
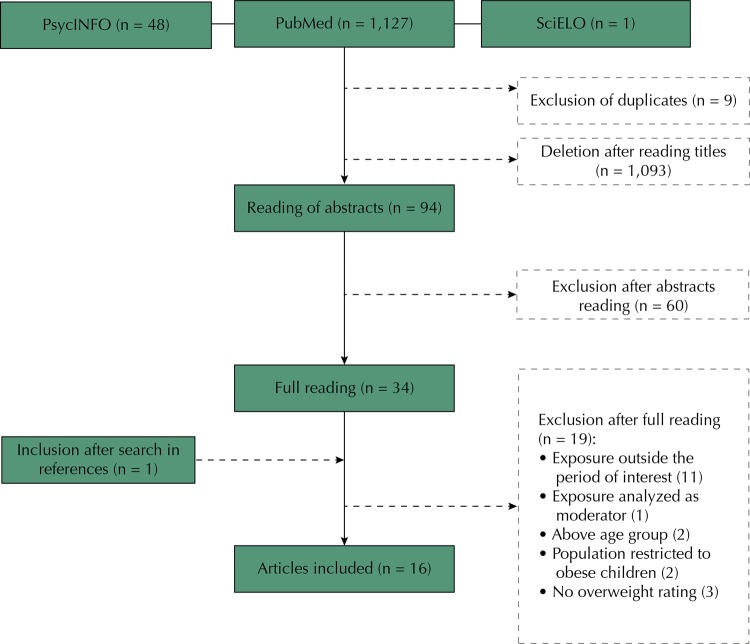
Flowchart of the systematic review.

Figure 1 presents the flowchart of the selection steps of articles.

Although the bibliographic search covered mothers and fathers, no studies evaluating both or only fathers were found, a fact that restricted this review to the association between maternal mental health and overweight/obesity of children.

### Characteristics of the Studies Selected

Studies that met the inclusion criteria for this review were published between 2007 and 2019. From the total, five were carried out in middle-income countries^15^, four of them in Brazil and one in Mexico. Eleven were developed in high-income countries^9,12,20^, six of them conducted in the United States, two in Australia, two in the United Kingdom and one in Spain. One of the North American studies evaluated only mothers and children of Mexican descent^21^.

Regarding the design, in studies developed in middle-income countries, two were longitudinal^15,18^ and three cross-sectional^16,17,19^. In high-income countries, six of the studies found were longitudinal^20^, one control case^9^ and four cross-sectional^12,24,25,27^.

In relation to the study populations, generally, longitudinal and transverse samples evaluated population-based samples. The smallest number of participants was 159 individuals^26^ and the largest was more than 10,000 mother-child pairs^24^. The only case-control study examined, in total, 100 mother-child pairs^9^.

Symptoms of maternal depression and anxiety were measured during the pregnancy and childhood of the children in only one study^26^, whereas the others were limited to childhood, ranging between the first months after childbirth to approximately the age of 12. In longitudinal studies, the measurements of such disorders were performed mostly two to four times; however, in O’Brien’s et al.^20^ work, in which ecological factors for the development of obesity in childhood were investigated, maternal depressive symptoms were measured nine times (from six months to 11 years old of children).

The evaluation of these mental disorders occurred in diverse ways, with different instruments used to detect maternal symptoms of depression and anxiety. Out of the 16 studies, only three evaluated anxiety symptoms, in addition to the depressive ones^9,25,27^. In some cases, more than one scale was used in the same study, according to the most indicated for the measurement point. Most of them applied the Center for Epidemiological Studies Depression Scale (CES-D)^16^. In two of them, in addition to CES-D, other scales were used, depending on the moment of measurement: Edinburgh Postnatal Depression Scale (EPDS)^18^ and Depression Scale of the Composite International Diagnostic Interview-Short Form (CIDI-SF)^21^. EPDS was used uniquely in two other surveys^15,19^. For the evaluation of maternal depressive symptoms and anxiety, two studies used the Depression, Anxiety and Stress Scale – 21 Items (DASS-21)^26,27^ and one study evaluated them with two instruments separately: Beck Depression Inventory-II *(*BDI) for depressive symptoms and The State Trait Anxiety Inventory (STAI) to assess trait and state of anxiety^8^. Two studies used the scale to assess psychological distress Kessler Psychological Distress Scale (K-6)^24^, whereas only one study used the Patient Health Questionnaire (PHQ-9)^25^ and another, the depression subscale of the Brief Symptom Inventory-18 (BSI-18)^12^.

For the assessment of child nutritional status, 14 of the 16 studies used body mass index (BMI) according to age and sex. Only two studies used different instruments: one used the weight for age index (W/A)^15^ and the other, the weight for length index (W/L)^16^ of the children. In most studies, anthropometric measurements were performed by trained interviewers, but two studies do not report the method^9,19^and, in one study, the measurements were taken from medical records^25^.

In three studies, nutritional status was analyzed longitudinally^21^, with children aged between nine months and 12 years as their study population. In the rest of them, the outcome was evaluated once, including children aged between six months and 12 years, although most of them were investigated during early childhood and preschool age.

The most used socioeconomic and maternal adjustment variables were schooling, family income, BMI or pre-gestational weight, marital status, employment status and family structure, considering that three or fewer studies also adjusted for other variables, such as: current, previous or during pregnancy smoking; maternal age; ethnicity; parity; socioeconomic status; family support; health insurance; number of residents at home; sanitation scale; use of health services; chronic disease; participation in activities with the child and in the Family Health Program. The most frequently used child-related variables were sex and ethnicity, few studies included birth weight, duration of breastfeeding, single child, age, prematurity, intrauterine growth restriction, recent disease, time in each parent’s households when those are divorced, sleep and screen time, introduction of solid foods and food insecurity.

Three studies performed analyses considering mediating variables. Hope et al.^28^ considered mediators diet and physical activity of the child, which mitigated risks, but maintained the association between maternal mental disorders, especially with severe symptoms, and childhood obesity. The association lost significance only in the complete model, considering the confounding and mediation variables. The work of Blanco et al.^9^ found indirect effects on the association between maternal depression or anxiety and childhood obesity, with emotional over-involvement and maladaptive coping. McConley et al.^12^ showed an association between maternal depression and single parenthood with childhood obesity, mediated by the quality of parenthood and its relationship with leisure activities and sedentary behavior of children. The studies demonstrated effects of small magnitude (effect sizes described in Chart 1), suggesting that other factors influence the etiology of childhood obesity.

**Chart 1:** Brief description of the articles selected in the literature review on the association between parental mental health and childhood obesity (n = 16).

Author and year, location, sample and design	Main objective	Exhibitions and instrument	Outcome and analysis (covariates)	Main results
Hope, 2019 United Kingdom n = 9,206 Cohort	To determine whether children’s exposure to average or severe maternal mental distress at age of 5 was associated with increased risks of overweight and obesity at age of 11.	Maternal mental disorder (average ≥ 4, severe ≥ 13) (5 years after childbirth) Kessler Psychological Distress Scale (Kessler-6)	BMI (normal, overweight, obese) 11 years old Multinomial logistic regression (sex and ethnicity of the child, maternal BMI, smoking during pregnancy, birth weight, breastfeeding time, food introduction, maternal education, family structure, income, mother’s participation in activities with the child, child’s sleep and screen time; mediators: dietary factors and physical activity of the child)	- The risk of obesity at age 11 increased with the severity of maternal mental distress at age 5: RRR (moderate) = 1.43; 95%CI 1.17–1.75 and RRR (severe) = 2.27; 95%CI 1.42–3.63. - The adjustment for each set of explanatory factors (particularly the first years and sociodemographic confounding factors) reduced but did not eliminate these high risks. - In the fully adjusted model, the risks were mitigated to non-significance: RRR (moderate) = 1.14; 95%CI 0.92–1.41 and RRR (severe) = 1.26; CI95% 0.75–2.11.
Lima, 2017 Brazil n = 1,381 Cohort	To check associations between maternal depressive symptoms with childhood malnutrition or overweight.	Maternal depression (≥ 22; ≥ 12) (prenatal, 12 and 24 months) Center for Epidemiologic Studies Depression Scale (CES-D) and *Edinburgh Postnatal Depression Scale* (EPDS)	BMIz (> 2 SD) 12–23 months Logistic regression (family income, schooling, number of children)	- No associations were found between MDS and childhood overweight (OR = 1.07; 95%CI 0.67–1.71).
Blanco et al., 2017 Spain n = 100 (50 cases, 50 controls) Case-control	To analyze the differences in maternal depression and anxiety, family functioning, expressed emotion and coping skills among mothers of children with obesity and normal weight.	Depression (≥ 20) and maternal anxiety (p > 75) (8–12 years of children) The State Trait Anxiety Inventory (STAI) Beck Depression Inventory-II (BDI)	BMIz (cases: p ≥ 97, controls: p < 85) 8-12 years old *Path analysis* Model of structural equations (adjusted for maternal BMI; model: critical comments, emotional overinvolvement, maladaptive coping)	- Mothers of obese children had a higher anxiety trait (z = -2.58; p = 0.01) and a tendency to significance for anxiety status (z = -1.85; p = 0.064). - There was an association between child’s obesity and risk of maternal depression (z = 4.39; p = 0.036). - In the trajectory analysis, maternal depression or anxiety did not directly predict the BMIz of the children (-0.13; p = 0.244). - Indirect effects were found due to the association between maternal depression or anxiety and emotional overinvolvement (indirect effect: 0.19; p = 0.006) and maladaptive coping (indirect effect: 0.09; p = 0.016).
de Castro et al., 2017 Mexico n = 4,240 Cross-sectional	To study the association of maternal depressive symptoms and early child health and development outcomes in mothers of low and high socioeconomic status.	Maternal depression (≥ 9) Center for Epidemiologic Studies Depression Scale (CES-D)	BMIz (> 2 SD) Under 5 years Wald for complex samples	- There was no significant difference for overweight of children between mothers with and without depressive symptoms (10.5% *versus* 9.72%, respectively; p = 0.66).
Audelo et al., 2016 USA (Mexican ethnicity) n = 332 Cohort	Prospectively investigate the association between depressive symptoms in women with children aged between 1 and 7 years, and childhood overweight and obesity at seven years in Latin families.	Maternal depression (moderate ≥ 16, severe ≥ 20) (1; 3,5 and 7years of children) Center for Epidemiologic Studies Depression Scale (CES-D)	BMIp (overweight ≥ 85, obesity ≥ 95) 7 years old Multiple logistic regression (number of years in the USA, pre-gestational weight, smoking during pregnancy, poverty status, housing density, social support, birth weight, food insecurity at 7 years)	- Children of women with recurrent depression (in 3 moments) were more likely to be overweight or obese at 7 years of age (OR = 2.4; 95%CI 1.1–5.6). - Maternal depression once or twice in thus period did not affect the child’s BMI.
Brentani, 2016 Brazil n = 798 Cross-sectional	To analyze the empirical relationship between maternal depression and development of 1-year-old children using cohort data.	Maternal depression (possible 10–13, probable ≥ 13) Edinburgh Postnatal Depression Scale (EPDS)	BMIz (> 2 SD) 12 months Multivariate linear regression (child: age, sex, twin, SGA, premature; mother: age, schooling, marital status, income)	- No association was found between maternal depression and obesity of children (delta = 0.012; p > 0.05).
Morrissey & Dagher, 2014 USA n = 6,500 Cohort	To study the early and contemporary associations between maternal depressive symptoms and children’s BMI, obesity and food intake.	Maternal Depression (≥ 9) (9 months; 2, 4 and 5.5 years of children) Center for Epidemiologic Studies Depression Scale (CES-D) 9 months, 4 and 5.5 years Depression Scale of the Composite International Diagnostic Interview Short Form (CIDI-SF): 2 years old	W/Lz (at 9 months) – (≥ p95) BMIz (2, 4 and 5.5 years) – (≥ p95) Linear model of probabilities (child’s age, sex, race/ethnicity, health insurance, pre-gestational weight, maternal and paternal working hours, family structure, poverty situation, urban or rural area; parents’ schooling)	- MDS were associated with a small decrease in the probability of the child being obese (0.8 percentage points). - Longer duration of maternal depressive symptoms was associated with higher BMI (0.02 SD) among children with parents without a university degree.
USA Cohort	To examine the association between maternal depressive symptoms during children’s early childhood and, later, overweight in childhood.	Maternal Depression (≥ 16) (1, 24 and 36 months of children) Center for Epidemiologic Studies Depression Scale (CES-D)	BMIp (≥ 85) 1st, 3rd and 6th grades Generalized estimation equation (child: birth weight, race, sex; maternal: race, age, schooling, work, marital status, family income, breastfeeding, smoking before pregnancy, social support)	- Children of depressed mothers in all three moments were more likely to be overweight than those of undepressed mothers: OR = 1.7; 95%CI 1.01–2.87 in the model adjusted for child characteristics and OR = 2.13; 95%CI 1.05-4.31 in the model adjusted for maternal characteristics.
Ramasubramanian, 2013 United Kingdom n = 10,465 Cross-sectional	To cross-sectionally examine the relationship between mother’s severe psychological distress and obesity in early childhood in a birth cohort.	Maternal mental disorder (≥ 13) (3 years after childbirth) Kessler Psychological Distress Scale (Kessler-6)	BMIp (≥ 95) 3 years old Multivariable logistic regression (SES, family support, schooling, maternal work, pre-gestational overweight, maternal chronic disease)	- Severe maternal distress suffering was associated with childhood obesity in crude analyses (OR = 1.62; 95%CI 1.11–2.37; p = 0.01) and adjusted (OR = 1.59; 95%CI 1.08–2.34; p = 0.01).
Gross, 2013 USA n = 401 Cross-sectional	To characterize the relationship between maternal depressive symptoms and the child’s weight, feeding practices that promote obesity and behaviors related to physical activity in low-income urban families.	Maternal depression (mild 5–9, moderate to severe 10–29) Patient Health Questionnaire-9 (PHQ-9)	BMIp (≥ 85) ≅ 5 years Multivariable logistic regression (sex, single child; health insurance, age, schooling, race, marital status and maternal employment status)	- Mothers with moderate to severe depressive symptoms were more likely to have overweight children than mothers without symptoms (adjusted OR = 2.62; 95%CI 1.02–6.70).
Gemmill, 2013 Australia n = 159 Cohort	To investigate whether prenatal and/or concomitant maternal depressive and anxious symptoms were predictive of the control of infant feeding practices and to examine whether the control of these practices predicted infant BMI.	Depression and Anxiety (NR) (pregnancy and 201507 years) Depression Anxiety Stress Scale (DASS-21)	BMI (NR) 2-7 years-old Multiple hierarchical regression (S1: family income, maternal education and age; S2: number of children and sex; S3: BMI in early childhood, concern with the child’s weight, depression, anxiety and stress)	- Maternal depression and anxiety were neither predictors of the child’s BMI (β = 0.24 and -0.14, respectively; p > 0.05), nor of the child’s maternal feeding practices (pressure to eat, restriction and monitoring). These were also not predictive of childhood BMI.
McConley, 2011 USA n = 4,601 Cross-sectional	To clarify the relationship between family structure, maternal depression and overweight of the child, and if it varies according to race/ethnicity or child’s sex.	Maternal depression (NR) Brief Symptom Inventory (BSI) depression subscale	BMIp (overweight 85–95, obesity > 95) 5th grade Modeling of structural equations (model: family structure, maternal depression, quality of parenthood, sedentary behavior, healthy food index, leisure activities, child’s BMI)	- The association between MDS and childhood BMI was mediated by the quality of parenthood (PE = -0.09) and its relationship with leisure activity (PE = -0.06), and children’s sedentary behavior (PE = 0.06) (p < 0.05).
Santos et al., 2010 Brazil n = 3,748 Cohort	To investigate the association between postpartum maternal depression and child growth at 4 years of age.	Maternal depression (≥ 13) (1, 2 and 4 years after childbirth) Edinburgh Postnatal Depression Scale (EPDS)	W/Az, (> 2 DP) 4 years old Multiple logistic regression (maternal age at birth, ethnicity, family income, education, marital status, smoking, pre-gestational BMI, use of health services)	- The effect of MDS in one or two of the three follow-ups (OR = 1.0; 95%CI 0.8–1.3) or all three (OR = 1.6; 95%CI 1.0–2.5) was not associated with childhood obesity.
Surkan et al., 2008 Brazil n = 589 Cross-sectional	To evaluate whether maternal depressive symptoms are associated with overweight in children aged between 6 and 24 months	Maternal depression (≥ 16) (6 to 24 months after childbirth) Center for Epidemiologic Studies Depression Scale (CES-D)	W/Lz (overweight ≥ p85, obesity > p95) 6 to 24 months old Multiple logistic regression (sex, birth weight, age, duration of breastfeeding, maternal education; sanitation scale, SES, number of children at home; recent child disease and participation in the Family Health Program)	- Overweight and childhood obesity were higher in children of mothers with high depressive symptoms than in those of asymptomatic mothers (39.9% *versus* 28.5%; p = 0.004; and 20.9% *versus* 10.3%; p = 0.0005, respectively). - Children of mothers with high depressive scores were almost twice as likely to be overweight (OR = 1.7; 95%CI 1.4–2.2) and obese (OR = 2.3; 95%CI 1.6–3.3).
O’Brien, 2007 USA n = 653 Cohort	To investigate ecological correlates of the development of overweight in a sample of children followed for 12 years.	Maternal depression (NR) (6, 15, 24, 36 and 54 months after childbirth and in the 1st, 3rd, 5th and 6th grade of the children) Center for Epidemiologic Studies Depression Scale (CES-D)	BMIp (≥ 85) 24, 36 and 54 months; 1st, 3rd, 5th and 6th grades Multiple logistic regression (sex and ethnicity of the child, maternal education, family income, marital status, time the child lives in households of divorced parents)	- Maternal depression did not differ between growth groups (never overweight, preschool overweight, school overweight and return to normal weight [OR - 1.0; p > 0.05]) and it was not a predictor of belonging to a specific group.
Gibson, 2007 Australia n = 329 Cross-sectional	To investigate the relationship between child’s weight and a wide range of family and maternal factors.	Depression and Anxiety (NR) Depression Anxiety Stress Scale (DASS-21)	BMIz (NR) 6–13 years old Multivariable linear regression (maternal BMI, schooling, income, family structure, number of residents at home, sex)	- Childhood obesity was not associated with depression (β = 0.01 [95%CI - 0.02; 0.04]) and maternal anxiety (β = 0.02 [95%CI -0.06; 0.06]).

BMI: body mass index; BMIz: BMI z-score; BMIp: BMI percentile; NR: non-reported; RRR: relative risk ratio; SD: standard deviation; SGA: small for gestational age; 95%CI: 95% confidence interval; MDS: maternal depressive symptom; OR: odds ratio; SE: standardized estimation; W/Lz: weight to length z-score; W/Az: weight-for-age z-score; SS: socioeconomic status

### Main Findings

Regarding the main results related to the association between maternal depressive or anxiety symptoms and overweight in children, we observed that, out of the 16 studies, eight found a positive association between exposure and outcome of interest, one conducted in Brazil^16^ and seven in high-income countries^9,12,21^. Of the latter, association between maternal depressive symptoms and children’s BMI varied according to the chronicity of the symptoms. Three of these studies^21^, all conducted in the United States, found an association with their children’s overweight only when chronicity of maternal depressive symptoms was present (positive measures for symptoms in three follow-ups). However, Morrissey et al.^22^ observed this association only in less educated mothers (without higher education) and an inverse association with the more educated ones.

Chart 1 presents the synthesis of the articles included in this systematic review.

## DISCUSSION

### Main Findings

This systematic review identified 16 studies evaluating the association between maternal symptoms of depression and/or anxiety and childhood obesity in childhood and found a positive relationship in half of them. This result seems to have been influenced by the quality of parenting of mothers with depressive or anxious symptoms, that is, by the way functions and activities are developed by them. Mothers with depressive symptoms may have lower parental quality, i.e. lower ability to perceive and to interpret signals and communications expressed in their children’s behavior, such as less care with meal rules and routines and attention to the quality and quantity of the food consumed^5,29^. The low control of children’s diet can lead them to consume less healthy foods^22^ when younger and/or allow unhealthy food choices when they are older, factors that corroborate with greater weight gain^30,31^.

Moreover, other behaviors developed and related to maternal depressive/anxiety symptoms may explain the offspring overweight, among them: less control over sleep time^25,29^, lower level of physical activity^27^ and longer screen time^12,20,27^. In this sense, some of the studies in this review found a positive association mediated by variables of parental style and child behavior, such as feeding response capacity^26^, shorter sleep time^25^, expressed emotion and maladaptive coping^9^, restriction and monitoring of children’s feeding^25,26^, use of food as a reward ^25^ and lower physical activity of children^12,25,27^.

These practices, which involve direct and active participation of the mother, can be negatively affected by depression due to symptoms such as loss of interest, low energy and fatigue^25,32^. Depressed mothers are generally less responsive to their child and choose coping strategies that require less cognitive effort^3,32^. Permissive parenting, which occurs when the mother places few demands and fails to establish limits on child’s behavior, has been positively associated with childhood obesity^3,12,32^. Children of depressed mothers present higher failure rates in weight intervention programs^33^ signaling that screening and treatment of maternal depression can improve outcomes related to the nutritional status of children.

Out of the total articles included in this review, five were performed in middle-income countries and only one of them, with a cross-sectional design, found an association between maternal depressive symptoms and overweight of children (between 6 and 24 months of age)^16^. Since low-income families present higher risk of both obesity^1^ and maternal depression^3^, a possible explanation for the non-association may be the interaction between socioeconomic and sociocultural factors in determining the effect of maternal mental health on child nutrition^13,15,30^. That is, families with lower economic level, generally, present lower levels of knowledge as well as lower purchasing power, which would affect the adequate child’s nutrition^34^. Despite escaping the scope of this review, it is important to highlight that some studies have found a link between maternal depressive symptoms and low weight of the child^15^ or lower weight than children of mothers without depressive symptoms^22,31^. However, the relationship seems to occur mainly in lower-income countries or in socioeconomically deprived populations living in higher income countries.

The divergent results between the studies of this review may also reflect methodological differences. One of the methodological factors that varied between them was the moment of evaluation of the child’s exposure to maternal mental disorders, ranging between less than 1 year and 12 years. This variation may have interfered with the results in two manners: (1) there may be an age window sensitive to exposure to maternal disorder for further development of obesity, and studies that did not include this sensitive period may not have found an association; (2) this association may be affected by the chronicity of exposure, and the effect would manifest when long exposure to maternal disorders occurs. Corroborating the limitation for the comparison between studies, the period in which maternal symptoms of depression and anxiety were measured did not present a pattern consistent with the BMI measurement of the children. It is not clear which would be the most specific time to measure maternal depressive symptoms to better predict the risk of overweight of children^11,35^.

### Limitations of the Studies

Within the limitations, seven authors indicated the losses that occurred in studies with longitudinal design, which often could not be classified^12,15,16,21^. The possibility of non-response bias was also considered^12,22,27^. These biases may affect the outcome of the studies, since mothers with severe depressive symptoms are more likely not to participate or to abandon the study, decreasing the magnitude or even suppressing the association. Another limitation indicated refers to the attribution of causality, inherent to cross-sectional studies, when the measurements of depressive symptoms occurred concomitantly with the performance of the child’s anthropometry^8,16,24,25^. Therefore, just as maternal mental disorder can affect the child’s weight, mothers of overweight children may have depressive symptoms as a result of this condition, making it impossible to infer causally from the relationship. Despite these limitations, the results of the studies were not invalidated, as the authors used techniques to minimize these problems, such as imputation of missing data and adequate statistical analyses, considering and discussing the limitations in their work.

The fact that information was reported by mothers may have impaired the validity of such data, since those with depressive symptoms are less likely to monitor the activities and behaviors of children. However, it is not clear whether maternal depressive symptoms would lead to excess or to underreport of such routines and behaviors. The focus of the studies on maternal depression was also indicated as a possible source of information error, correlated with the social desirability bias^28^.

Some authors indicated the lack of data on maternal BMI and absence or low quality of information on the child’s diet as limitations^12,16^. The first may confuse the association or be on the causal path between maternal mental disorder and the child’s weight, whereas the second may be a mediator of the association. Limitations in the measures of food consumption were also considered, in some cases explaining the lower magnitude^16,22^ or even the absence of association^18,20,27^. Some reasons discussed for the non-association included convenience sample^18^, losses on follow-up ^18,20,28^ and possible bias in reporting depressive symptoms^18,28^, as well as the possibility of residual confusion^15^. As discussed, these factors may have made the positive result of the association in these studies unfeasible.

Only three studies were found addressing maternal anxiety symptoms, along with depressive ones, which could help in a better understanding of this relationship, since anxious conditions may progress to depression or coexist with it. Only in one of these studies the association was significant^8^, but anxiety and depression were grouped into a single construct defined as maternal psychopathology, making it impossible to attribute the association to each disorder individually.

### Strengths of the Studies

Among the strengths of the methodology of the studies, we highlight the large number of individuals surveyed, some of them with national representativeness^12,16,24,28^. As a positive point, most of them collected anthropometric data by trained and standardized interviewers. Longitudinal studies allow establishing a temporal relationship between symptoms of maternal depression and childhood obesity, in addition to the measurement of this symptomatology in many follow-ups, being possible to distinguish between chronic symptoms and episodic ones.

Methodologically, this study sought research that collected information on maternal mental disorders during children’s childhood, since early childhood (two to five years of age) was identified as a critical period, in which behaviors (physical activity and diet) that influence obesity^36^are established. This review sought to discuss the results considering that the studies were conducted with populations from different places, factor that may influence the presence or absence of the association and that, consequently, must be weighed in the comparison of results. The heterogeneity between the studies regarding the measurement and categorization of maternal depression and child’s age at exposure in the outcome made it impossible to demonstrate a combined estimate of the effects in meta-analysis, which could better explain the results. Regarding the quality of the selected studies, most presented a good performance in the evaluation performed, and only two^9,19^ obtained scores below 10 points, out of a total of 15.

In the same sense of the results described, previous reviews^10,11,35^ found a positive relationship between depression^11,35^ and other maternal psychopathologies^11^ and obesity in children, with results diverging according to the measure of maternal disorders (pre or postnatal, in isolation or longitudinal) and with the different age groups of children (preschoolers, childhood and adolescence). The studies were conducted in 2012, 2014 and 2015 and included, respectively, 5, 9 and 20 articles. Although they present some differences regarding the inclusion criteria, they highlight the growing interest in the subject in recent years.

### Final Remarks

Despite the positive association in most studies, it is necessary to consider disagreements between the results observed, indicating that maternal mental health contributes to the promotion of childhood obesity, but it acts in association with other factors involved in its etiology. For a better understanding of this phenomenon, it is also important to investigate paternal and familial risk factors, such as mental health.

Some coping measures to the exposed problem are recommended, such as screening for maternal mental disorders since prenatal care and, after childbirth, in child’s routine consultations. In countries with greater social vulnerability with home health teams, diagnosis and treatment of these disorders can be more easily provided by health professionals’ training. Community health interventions in low- and middle-income countries including a maternal mental health component (home visit, empathic listening and cognitive-behavioral techniques) demonstrated success in the decline of depressive symptoms in women in the intervention group^37^. Based on this review, we conclude that evidence of a relationship between symptoms of maternal chronic depression and children’s excess weight exists, mediated by the behaviors of the mother and child. Therefore, it is essential to deepen knowledge about the mechanisms that influence this association so that policies of mental health care of women can be developed, indirectly impacting the nutritional status of children.

## References

[1] 1. NCD Risk Factor (NCD-RisC). Ezzati, M, Bentham, J, Di Cesare, M, Bilano, V, Bixby, H, Zhou, B, et al. Worldwide trends in body-mass index, underweight, overweight, and obesity from 1975 to 2016: a pooled analysis of 2416 population-based measurement studies in 128.9 million children, adolescents, and adults. Lancet. 2017;390(10113):2627-42. 10.1016/S0140-6736(17)32129-3 PMC5735219 29029897

[2] 2. World Health Organization. Depression and other common mental disorders: global health estimates. Geneva: WHO; 2017 [cited 2018 Nov 12]. Available from: https://www.who.int/mental_health/management/depression/prevalence_global_health_estimates/en/

[3] 3. World Health Organization. Maternal mental health and child health and development in low and middle income countries: report of the meeting held in Geneva, Switzerland, 30 January - 1 February, 2008. [cited 2018 Nov 12]. Available from: http://www.who.int/mental_health/prevention/suicide/mmh_jan08_meeting_report.pdf?ua=1

[4] 4. World Health Organization. Obesity: preventing and managing the global epidemic: report of a WHO Consultation. Geneva: WHO; 1998 [cited 2018 Nov 12]. (WHO Technical Report Series, 894). Available from: http://www.who.int/nutrition/publications/obesity/WHO_TRS_894/en/ 11234459

[5] 5. Russell CG, Russell A. A biopsychosocial approach to processes and pathways in the development of overweight and obesity in childhood: Insights from developmental theory and research. Obes Rev. 2019;20(5):725-49. 10.1111/obr.12838 30768750

[6] 6. Ventura AK, Birch LL. Does parenting affect children’s eating and weight status? Int J Behav Nutr Phys Act. 2008;5:15. 10.1186/1479-5868-5-15 PMC2276506 18346282

[7] 7. Kremers S, Sleddens E, Gerards S, Gubbels J, Rodenburg G, Gevers D, et al. General and food-specific parenting: measures and interplay. Child Obes. 2013;9 Suppl 1:S22-31. https://doi.or/10.1089/chi.2013.0026 10.1089/chi.2013.0026 PMC3746240 23944921

[8] 8. Park H, Sundaram R, Gilman SE, Bell G, Louis GMB, Yeung EH. Timing of maternal depression and sex-specific child growth, the Upstate KIDS Study. Obesity (Silver Spring). 2018;26(1):160-6. 10.1002/oby.22039 PMC5739947 29090856

[9] 9. Blanco M, Sepulveda AR, Lacruz T, Parks M, Real B, Martin-Peinador Y, et al. Examining maternal psychopathology, family functioning and coping skills in childhood obesity: a case-control study. Eur Eat Disord Rev. 2017;25(5):359-65. 10.1002/erv.2527 28568706

[10] 10. Benton PM, Skouteris H, Hayden M. Does maternal psychopathology increase the risk of pre-schooler obesity? A systematic review. Appetite. 2015;87:259-82. 10.1016/j.appet.2014.12.227 25572134

[11] 11. Milgrom J, Skouteris H, Worotniuk T, Henwood A, Bruce L. The association between ante- and postnatal depressive symptoms and obesity in both mother and child: a systematic review of the literature. Womens Health Issues. 2012;22(3):e319-28. 10.1016/j.whi.2011.12.001 22341777

[12] 12. McConley RL, Mrug S, Gilliland MJ, Lowry R, Elliott MN, Schuster MA, et al. Mediators of maternal depression and family structure on child BMI: parenting quality and risk factors for child overweight. Obesity (Silver Spring). 2011;19(2):345-52. 10.1038/oby.2010.177 20798670

[13] 13. Topham GL, Page MC, Hubbs-Tait L, Rutledge JM, Kennedy TS, Shriver L, et al. Maternal depression and socio-economic status moderate the parenting style/child obesity association. Public Health Nutr. 2010;13(8):1237-44. 10.1017/S1368980009992163 19968899

[14] 14. Downs SH, Black N. The feasibility of creating a checklist for the assessment of the methodological quality both of randomised and non-randomised studies of health care interventions. J Epidemiol Community Health. 1998;52(6):377-84. 10.1136/jech.52.6.377 PMC1756728 9764259

[15] 15. Santos IS, Matijasevich A, Domingues MR, Barros AJD, Barros FCF. Long-lasting maternal depression and child growth at 4 years of age: a cohort study. J Pediatr. 2010;157(3):401-6. 10.1016/j.jpeds.2010.03.008 PMC2937222 20400093

[16] 16. Surkan PJ, Kawachi I, Peterson KE. Childhood overweight and maternal depressive symptoms. J Epidemiol Community Health. 2008;62(5):e11. 10.1136/jech.2007.065664 18431837

[17] 17. Castro F, Place JM, Villalobos A, Rojas R, Barrientos T, Frongillo EA. Poor early childhood outcomes attributable to maternal depression in Mexican women. Arch Womens Ment Health. 2017;20(4):561-8.. 10.1007/s00737-017-0736-7 28601985

[18] 18. Lima TFM, Maciel WM, Alencar MN, Cruz JAS, Carvalho CA, Silva AAM. Associação entre sintomas depressivos maternos com desnutrição ou excesso de peso infantis. Rev Bras Saude Mater Infant. 2017;17(3):603-13. 10.1590/1806-93042017000300010

[19] 19. Brentani A, Fink G. Maternal depression and child development: evidence from Sao Paulo’s Western Region Cohort Study. Rev Assoc Med Bras. 2016;62(6):524-9. 10.1590/1806-9282.62.06.524 27849229

[20] 20. O’Brien M, Nader PR, Houts RM, Bradley R, Friedman SL, Belsky J, et al. The ecology of childhood overweight: a 12-year longitudinal analysis. Int J Obes (Lond). 2007;31(9):1469-78. 10.1038/sj.ijo.0803611 PMC2137173 17406272

[21] 21. Audelo J, Kogut K, Harley KG, Rosas LG, Stein L, Eskenazi B. Maternal depression and childhood overweight in the CHAMACOS Study of Mexican-American Children. Mater Child Health J. 2016;20(7):1405-14. https://doi.org;10.1007/s10995-016-1937-9 10.1007/s10995-016-1937-9 27007986

[22] 22. Morrissey TW, Dagher RK. A longitudinal analysis of maternal depressive symptoms and children’s food consumption and weight outcomes. Public Health Nutr. 2014;17(12):2759-68. 10.1017/S1368980013003376 PMC10282476 24476574

[23] 23. Wang L, Anderson JL, Dalton Iii WT, Wu T, Liu X, Zheng S, et al. Maternal depressive symptoms and the risk of overweight in their children. Matern Child Health J. 2013;17(5):940-8. 10.1007/s10995-012-1080-1 22833333

[24] 24. Ramasubramanian L, Lane S, Rahman A. The association between maternal serious psychological distress and child obesity at 3 years: a cross-sectional analysis of the UK Millennium Cohort Data. Child Care Health Dev. 2013;39(1):134-40. 10.1111/j.1365-2214.2011.01325.x 22040298

[25] 25. Gross RS, Velazco NK, Briggs RD, Racine AD. Maternal depressive symptoms and child obesity in low-income urban families. Acad Pediatr. 2013;13(4):356-63. 10.1016/j.acap.2013.04.002 23830021

[26] 26. Gemmill AW, Worotniuk T, Holt CJ, Skouteris H, Milgrom J. Maternal psychological factors and controlled child feeding practices in relation to child body mass index. Child Obes. 2013;9(4):326-37. 10.1089/chi.2012.0135 23782306

[27] 27. Gibson LY, Byrne SM, Davis EA, Blair E, Jacoby P, Zubrick SR. The role of family and maternal factors in childhood obesity. Med J Aust. 2007;186(11):591-5. 10.5694/j.1326-5377.2007.tb01061.x 17547550

[28] 28. Hope S, Pearce A, Chittleborough C, Deighton J, Maika A, Micali N, et al. Temporal effects of maternal psychological distress on child mental health problems at ages 3, 5, 7 and 11: analysis from the UK Millennium Cohort Study. Psychol Med. 2019;49(4):664-74. 10.1017/S0033291718001368 PMC6378410 29886852

[29] 29. McCurdy K, Tovar A, Kaar JL, Vadiveloo M. Pathways between maternal depression, the family environment, and child BMI Z scores. Appetite. 2019;134:148-54. 10.1016/j.appet.2018.12.010 30599152

[30] 30. McCurdy K, Gorman KS, Kisler T, Metallinos-Katsaras E. Associations between family food behaviors, maternal depression, and child weight among low-income children. Appetite. 2014;79:97-105. 10.1016/j.appet.2014.04.015 PMC4096897 24768937

[31] 31. Duarte CS, Shen S, Wu P, Must A. Maternal depression and child BMI: longitudinal findings from a US sample. Pediatr Obes. 2012;7(2):124-33. 10.1111/j.2047-6310.2011.00012.x PMC4353610 22434752

[32] 32. Lovejoy MC, Graczyk PA, O’Hare E, Neuman G. Maternal depression and parenting behavior: a meta-analytic review. Clin Psychol Rev. 2000;20(5):561-92. 10.1016/s0272-7358(98)00100-7 10860167

[33] 33. Frohlich G, Pott W, Albayrak O, Hebebrand J, Pauli-Pott U. Conditions of long-term success in a lifestyle intervention for overweight and obese youths. Pediatrics. 2011;128(4):e779-85. 10.1542/peds.2010-3395 21911346

[34] 34. Harrison K, Bost KK, McBride BA, Donovan SM, Grigsby-Toussaint DS, Kim J, et al. Toward a developmental conceptualization of contributors to overweight and obesity in childhood. The Six-Cs Model. Child Dev Perspect. 2011;5(1):50-8. 10.1111/j.1750-8606.2010.00150.x

[35] 35. Lampard AM, Franckle RL, Davison KK. Maternal depression and childhood obesity: a systematic review. Prev Med. 2014;59:60-7. 10.1016/j.ypmed.2013.11.020 PMC4172574 24291685

[36] 36. Jones RA, Okely AD, Caputi P, Cliff DP. Relationships between child, parent and community characteristics and weight status among young children. Int J Pediatr Obes. 2010;5(3):256-64. 10.3109/17477160903271971 19900149

[37] 37. Baker-Henningham H, Powell C, Walker S, Grantham-McGregor S. The effect of early stimulation on maternal depression: a cluster randomised controlled trial. Arch Dis Child. 2005;90(12):1230-4. 10.1136/adc.2005.073015 PMC1720239 16159905

[38] 38. Rahman A, Patel V, Maselko J, Kirkwood B. The neglected ‘M’ in MCH programmes: why mental health of mothers is important for child nutrition. Trop Med Int Health. 2008;13(4):579-83. 10.1111/j.1365-3156.2008.02036.x 18318697

[39] 39. Cooper PJ, Tomlinson M, Swartz L, Landman M, Molteno C, Stein A, et al. Improving quality of mother-infant relationship and infant attachment in socioeconomically deprived community in South Africa: randomised controlled trial. BMJ. 2009;338:b974. 10.1136/bmj.b974 PMC2669116 19366752

