# Supplementary material for: Systematic review: Symptoms of parental depression and anxiety and offspring overweight
Source: Rev Saude Publica. 2020 May 15;54:49. doi: 10.11606/s1518-8787.2020054001731 (PMC7234214; doi:10.11606/s1518-8787.2020054001731)
Supplement: Translation

# Translation: Revisão sistemática: Sintomas de depressão e ansiedade parental e excesso de peso da prole

**Authors:** Paula Lobo Marco, Inaê Dutra Valério, Christian Loret de Mola Zanatti, Helen Gonçalves

**Affiliations:** I Brasil Universidade Federal de Pelotas. Faculdade de Medicina. Departamento de Medicina Social. Pelotas, RS, Brasil; II Brasil Universidade Federal do Rio Grande. Faculdade de Medicina. Programa de Pós-graduação em Saúde Pública. Departamento de Medicina Social. Pelotas, RS, Brasil

**Keywords:** Relações Mãe-Filho, Transtornos do Humor, Transtornos de Ansiedade, Obesidade Pediátrica, Revisão Sistemática

## Abstract

**OBJETIVO:**

Avaliar a literatura existente acerca da associação entre depressão e ansiedade dos pais e sua influência no excesso de peso dos filhos durante a infância, identificando possíveis mecanismos envolvidos nessa associação.

**MÉTODOS:**

Foi realizada uma busca na literatura, de forma sistemática, nas bases de dados PubMed, PsycINFO e SciELO, usando os descritores: (maternal OR mother* OR parent* OR paternal OR father) AND (“common mental disorder” OR “mental health” OR “mental disorder” OR “depressive disorder” OR depress* OR anxiety OR “anxiety disorder”) AND (child* OR pediatric OR offspring) AND (overweight OR obes* OR “body mass index” OR BMI). Foram encontrados 1.187 artigos após seleção por pares.

**RESULTADOS:**

Foram selecionados 16 artigos que atingiram os critérios para inclusão na revisão. A maioria investigou sintomas depressivos e somente três, sintomas ansiosos maternos. Os estudos avaliados mostraram resultados sugestivos de associação positiva entre sintomas de depressão materna e maior risco de excesso de peso nos filhos. Os resultados divergiram de acordo com a cronicidade dos sintomas depressivos (depressão episódica ou recorrente) e renda do país investigado (alta ou média renda). Foram identificados mecanismos que passam pela qualidade da parentalidade, afetando comportamentos relacionados à atividade física e alimentação da criança, como mediadores da associação.

**CONCLUSÕES:**

Concluímos que há evidências de uma relação positiva entre a ocorrência de sintomas maternos de depressão e ansiedade e o excesso de peso dos filhos. Ressalta-se a necessidade de uma melhor compreensão do impacto do momento de ocorrência dos sintomas depressivos e dos fatores contextuais envolvidos nessa relação para que possam ser implementadas estratégias de intervenção eficazes.

## INTRODUÇÃO

Nas últimas quatro décadas, o número de crianças e adolescentes obesos aumentou dez vezes no mundo, de 11 milhões em 1975 para 124 milhões em 2016^1^. Segundo essa tendência, até 2022 a obesidade ultrapassará a desnutrição nestas fases da vida^1^.

Além do aumento da obesidade na população de crianças e adolescentes, outro fenômeno global contemporâneo é a elevada prevalência dos distúrbios de depressão e ansiedade notada entre jovens e adultos^2^. Em torno de 4,4% da população mundial está deprimida e 3,6% com distúrbios de ansiedade, alcançando, respectivamente, taxas de 5,9% e 7,7% na região das Américas^2^. Entre 2005 e 2015, constatou-se um aumento mundial de 18% na depressão e de 15% nos distúrbios de ansiedade. Apesar das diferenças entre países, em todos há uma maior prevalência de ambos os transtornos entre as mulheres^3^.

A obesidade é uma doença com etiologia multifatorial^4^, incluindo fatores biológicos, como a genética; fatores ambientais, como a exposição a ambientes obesogênicos; e fatores psicossociais que envolvem a alimentação, como a qualidade da parentalidade^5,6^. No tocante à alimentação, a parentalidade se manifesta por meio de práticas como controle alimentar da criança e do uso de alimentos como recompensa, sendo associada ao peso na infância^6,7^.

Um fator que influencia diretamente na qualidade da parentalidade e, como consequência, na saúde dos filhos, é a presença de transtornos mentais nos pais^8^. Mães com depressão, em geral, têm problemas para prover cuidados adequados aos filhos, afetando seu nível de responsividade em relação à criança^7^. Isso reflete-se em hábitos alimentares menos saudáveis e menor controle sobre o tempo de visualização de televisão e outros comportamentos sedentários, com baixo estímulo a atividades recreativas que necessitem de envolvimento materno direto^9,10^.

Alguns estudos demonstraram uma relação entre transtornos mentais das mães e estado nutricional dos filhos^10^; no mesmo sentido, foi encontrado que mulheres deprimidas no pós-natal tendem a amamentar por menos tempo que o recomendado^13^ e a ter filhos desnutridos ou obesos^8^. Devido às taxas crescentes de transtornos depressivos e ansiosos, torna-se de grande relevância a investigação das suas consequências na relação pais-filhos, especialmente durante a infância, período quando são formados os hábitos da criança.

Esta revisão sistemática da literatura objetivou avaliar estudos acerca da associação entre os transtornos de ansiedade e depressão parentais e sua influência no excesso de peso dos filhos durante a infância, identificando possíveis mecanismos envolvidos nessa associação.

## MÉTODOS

### Delineamento do Estudo

Foi conduzida uma revisão sistemática da literatura com o objetivo de identificar artigos originais avaliando a associação entre os transtornos de depressão e ansiedade parentais e excesso de peso dos filhos entre 1 e 12 anos.

### Métodos de Busca e Seleção dos Estudos

A busca foi realizada entre novembro de 2018 e fevereiro de 2019, nas bases de dados PubMed, SciELO e PsycINFO. Os termos de busca utilizados foram: (maternal OR mother* OR parent* OR paternal OR father) AND (“common mental disorder” OR “mental health” OR “mental disorder” OR “depressive disorder” OR depress* OR anxiety OR “anxiety disorder”) AND (child* OR pediatric OR offspring) AND (overweight OR obes* OR “body mass index” OR BMI). A única restrição foi que os estudos fossem realizados em humanos. Além disso, foi realizada busca ativa nas referências dos artigos selecionados.

O processo de revisão foi realizado por dois revisores independentes (PLM e IDV), cujas discordâncias foram discutidas até o consenso sobre a inclusão do artigo para compor a revisão.

### Critérios de Elegibilidade

Para a inclusão nesta revisão, os artigos deveriam avaliar a exposição aos transtornos mentais comuns (quaisquer tipos de transtornos depressivos e/ou de ansiedade) dos pais, e o desfecho excesso de peso ou obesidade das crianças, definido nos estudos por meio de métodos acurados de avaliação nutricional.

Salienta-se que, apesar de a depressão na gestação e no pós-parto (definido para os fins desta revisão como o período do nascimento aos 12 meses de idade da criança) não ser o foco desse trabalho, estudos com essas exposições foram incluídos quando, ao analisarem o transtorno de forma longitudinal, as informações extrapolaram esse período.

### Critérios de Exclusão

Foram excluídos estudos que não tivessem medidas de depressão e ansiedade validadas, realizadas exclusivamente no período pré-natal, gestacional ou dentro de um ano após o parto, assim como os que avaliaram somente crianças no primeiro ano de vida, adolescentes, crianças com problemas de saúde que pudessem afetar o peso ou exclusivamente crianças obesas.

### Avaliação da Qualidade

Os artigos desta revisão foram avaliados quanto à adequação metodológica empregada na seleção da amostra, coleta e análise dos dados de acordo com o instrumento Downs & Black^14^ adaptado para estudos observacionais, com escala variando de 0 a 15 pontos. Foram analisados os seguintes aspectos principais de cada estudo: definição dos objetivos e medidas de desfecho; definição dos critérios de inclusão e exclusão, período e local do estudo, procedimentos de amostragem (população-alvo e descrição das etapas da amostra final), descrição e/ou relato de perdas, medidas de variabilidade (erro/desvio-padrão e intervalo de confiança), descrição dos métodos de mensuração do desfecho (treinamento de pessoal, calibração de instrumentos e roupas apropriadas) e análise estatística (estatística apropriada, controle para fatores de confusão e cálculo de poder).

## RESULTADOS

### Seleção dos Estudos

Foram encontrados 1.196 artigos, somando-se as três bases de dados consultadas, restando 1.187 após exclusão das duplicatas (n = 9). A estratégia de busca identificou 1.127 publicações na base PubMed, 48 na base PsycINFO e 21 na base SciELO. As selecionadas foram importadas para uma biblioteca EndNote^®^ e, após a leitura dos títulos, foram escolhidos 94 registros por consenso entre os revisores. O motivo principal para exclusão foi a não satisfação dos critérios de inclusão relativos ao desfecho ou exposição. A partir da leitura dos resumos, foi feita a seleção dos artigos para a leitura na íntegra. Nessa etapa, 60 artigos foram excluídos por não avaliar o desfecho de interesse, restando 34 para leitura completa. Após a leitura na íntegra, 19 artigos foram excluídos pelas seguintes razões: avaliação da exposição fora do período de interesse (n = 11), população composta somente por crianças obesas ou em risco de obesidade (n = 2), faixa etária superior à determinada (n = 2), transtornos mentais maternos analisados como variável moderadora (n = 1) e não utilização de ponto de corte para classificação do estado nutricional das crianças (n = 3). Um estudo foi incluído após busca ativa nas referências dos artigos selecionados, somando 16 artigos para inclusão nesta revisão bibliográfica.

A Figura 1 apresenta o fluxograma das etapas de seleção dos artigos.

**Figura 1:** Fluxograma da revisão bibliográfica.

Apesar de a busca bibliográfica ter abrangido mães e pais, não foram encontrados estudos avaliando ambos ou somente pais, fato que restringiu esta revisão à associação entre saúde mental materna e excesso de peso/obesidade da prole.

### Características dos Estudos Selecionados

Os estudos que atenderam aos critérios para inclusão nesta revisão foram publicados entre os anos de 2007 e 2019. Do total, cinco foram realizados em países de média renda^15^, sendo quatro deles no Brasil e um no México. Onze foram desenvolvidos em países de alta renda^9,12,20^, sendo seis deles conduzidos nos Estados Unidos, dois na Austrália, dois no Reino Unido e um na Espanha. Um dos estudos norte-americanos avaliou somente mães e filhos de ascendência mexicana^21^.

Quanto ao delineamento, nos estudos desenvolvidos em países de média renda, dois foram longitudinais^15,18^ e três transversais^16,17,19^. Em países de alta renda, seis dos estudos encontrados foram do tipo longitudinal^20^, um caso-controle^9^ e quatro transversais^12,24,25,27^.

Em relação às populações em estudo, de modo geral, os longitudinais e transversais avaliaram amostras de base populacional. O menor número de participantes foi de 159 indivíduos^26^ e o maior foi superior a 10 mil pares mãe-filho^24^. O único estudo caso-controle examinou, no total, 100 pares mãe-filho^9^.

Os sintomas de depressão e ansiedade maternos foram medidos na gestação e infância dos filhos em apenas um estudo^26^, enquanto os demais se limitaram à infância, variando desde os primeiros meses após o parto até aproximadamente os 12 anos das crianças. Nos estudos longitudinais, as medidas desses transtornos foram realizadas majoritariamente de duas a quatro vezes; no entanto, no trabalho de O’Brien et al.^20^, em que foram pesquisados fatores ecológicos para o desenvolvimento da obesidade na infância, os sintomas depressivos maternos foram medidos nove vezes (dos seis meses aos 11 anos dos filhos).

A avaliação desses transtornos mentais ocorreu de diversas formas, com diferentes instrumentos usados para detectar sintomas maternos de depressão e ansiedade. Dos 16 estudos, apenas três avaliaram a sintomatologia de ansiedade, além da depressiva^9,25,27^. Em alguns casos, mais de uma escala foi utilizada num mesmo estudo, de acordo com o mais indicado para o momento da medida. A maior parte deles aplicou a *Center for Epidemiological Studies Depression Scale* (CES-D)^16^. Em dois deles, além da CES-D, foram utilizadas outras escalas, dependendo do momento da medida: *Edinburgh Postnatal Depression Scale* (EPDS)^18^ e *Depression Scale of the Composite International Diagnostic Interview Short Form* (CIDI-SF)^21^. O EPDS foi utilizado de maneira única em outros dois inquéritos^15,19^. Para a avaliação dos sintomas depressivos e de ansiedade maternos, dois estudos utilizaram a *Depression, Anxiety and Stress Scale – 21 Items* (DASS-21)^26,27^ e um trabalho os avaliou com dois instrumentos separadamente: *Beck Depression Inventory-II (*BDI) para sintomas depressivos e *The State Trait Anxiety Inventory* (STAI) para avaliar traço e estado de ansiedade^8^. Dois estudos utilizaram a escala para avaliação do sofrimento psicológico *Kessler Psychological Distress Scale* (K-6)^24,28^, enquanto apenas um estudo utilizou o *Patient Health Questionnaire* (PHQ-9)^25^ e outro, a subescala de depressão do *Brief Symptom Inventory-18* (BSI-18)^12^.

Para a avaliação do estado nutricional infantil, 14 dos 16 estudos utilizaram o índice de massa corporal (IMC) de acordo com idade e sexo. Apenas dois estudos utilizaram instrumentos diferentes: um utilizou o índice de peso para a idade (P/I)^15^ e outro, o índice de peso para o comprimento (P/C)^16^ das crianças. Na maior parte dos estudos, as medidas antropométricas foram realizadas por entrevistadores treinados, porém dois trabalhos não relatam o método^9,19^e outro avaliou medidas obtidas de registros médicos^25^.

Em três estudos o estado nutricional foi analisado longitudinalmente^21^, tendo como população de estudo crianças com idades entre nove meses e 12 anos. No restante deles, o desfecho foi avaliado em apenas um momento, contemplando crianças com idade entre seis meses e 12 anos, embora a maior parte tenha sido investigada durante a primeira infância e na idade pré-escolar.

As variáveis de ajuste socioeconômicas e maternas mais utilizadas foram a escolaridade, renda familiar, IMC ou peso pré-gestacional, estado civil, status de emprego e estrutura familiar, sendo que três trabalhos ou menos ajustaram também para outras variáveis, como: tabagismo atual, anterior ou durante a gestação; idade materna; etnia; paridade; nível socioeconômico; suporte familiar; seguro de saúde; número de moradores em casa; escala de saneamento; uso de serviços de saúde; doença crônica; participação em atividades com o filho e no Programa Saúde da Família. As variáveis relativas à criança mais frequentemente utilizadas foram sexo e etnia, e poucos estudos incluíram peso ao nascer, duração da amamentação, filho único, idade, prematuridade, restrição de crescimento intrauterino, doença recente, tempo nas casas do pai ou mãe quando separados, tempo de sono e de tela, introdução de alimentos sólidos e insegurança alimentar.

Três estudos realizaram análises considerando variáveis mediadoras. Hope et al.^28^ consideraram os mediadores dieta e atividade física da criança, os quais atenuaram os riscos, porém mantiveram a associação entre transtornos mentais maternos, especialmente com sintomas severos, e obesidade dos filhos. A associação perdeu a significância apenas no modelo completo, considerando as variáveis de confusão e mediação. O trabalho de Blanco et al.^9^ encontrou efeitos indiretos na associação entre depressão ou ansiedade materna e obesidade dos filhos, por meio do superenvolvimento emocional e enfrentamento desadaptativo. McConley et al.^12^ mostraram associação da depressão materna e monoparentalidade com a obesidade dos filhos, mediada pela qualidade da parentalidade e sua relação com atividades de lazer e comportamento sedentário das crianças. Os estudos demonstraram efeitos de pequena magnitude (medidas de efeito descritas no Quadro 1), sugerindo que outros fatores exercem influência na etiologia da obesidade infantil.

**Quadro 1:** Descrição resumida dos artigos selecionados na revisão bibliográfica sobre associação entre saúde mental parental e excesso de peso dos filhos (n = 16).

Autor e ano, local, amostra e delineamento	Objetivo principal	Exposições e instrumento	Desfecho e análise (covariáveis)	Principais resultados
Hope, 2019 Reino Unido n = 9.206 Coorte	Determinar se a exposição das crianças a um sofrimento mental materno médio ou grave aos 5 anos estava associada a aumento dos riscos de sobrepeso e obesidade aos 11 anos.	Transtorno mental materno (médio ≥ 4, severo ≥ 13) (5 anos após o parto) Kessler Psychological Distress Scale (Kessler-6)	IMC (normal, sobrepeso, obeso) 11 anos Regressão logística multinomial (sexo e etnia da criança, IMC materno, tabagismo na gestação, peso ao nascer, tempo de amamentação, introdução alimentar, escolaridade materna, estrutura familiar, renda, participação da mãe em atividades com o filho, tempo de sono e de tela da criança; mediadores: fatores dietéticos e atividade física da criança)	- O risco de obesidade aos 11 anos aumentou com a gravidade do sofrimento mental materno aos 5 anos: RRR (moderado) = 1,43; IC95% 1,17–1,75 e RRR (grave) = 2,27; IC95% 1,42–3,63. - O ajuste para cada conjunto de fatores explicativos (particularmente os primeiros anos e fatores de confusão sociodemográficos) reduziu, mas não eliminou esses riscos elevados. - No modelo totalmente ajustado, os riscos foram atenuados até a não significância: RRR (moderado) = 1,14; IC95% 0,92–1,41 e RRR (grave) = 1,26; IC95% 0,75–2,11.
Lima, 2017 Brasil n = 1.381 Coorte	Verificar associações entre sintomas depressivos maternos com desnutrição ou excesso de peso infantis.	Depressão materna (≥ 22; ≥ 12) (pré-natal, 12 e 24 meses) *Center for Epidemiologic Studies Depression Scale* (CES-D) e *Edinburgh Postnatal Depression Scale* (EPDS)	IMCz (> 2 DP) 12–23 meses Regressão logística (renda familiar, escolaridade, número de filhos)	- Não foram encontradas associações entre SDM e excesso de peso infantil (OR = 1,07; IC95% 0,67–1,71).
Blanco et al., 2017 Espanha n = 100 (50 casos, 50 controles) Caso-controle	Analisar as diferenças na depressão e ansiedade materna, funcionamento familiar, emoção expressa e habilidades de enfrentamento entre mães de crianças com obesidade e com peso normal.	Depressão (≥ 20) e ansiedade materna (p > 75) (8–12 anos dos filhos) *The State Trait Anxiety Inventory* (STAI) *Beck Depression Inventory*-II (BDI)	IMCz (casos: p ≥ 97, controles: p < 85) 8–12 anos de idade *Path analysis* Modelo de equações estruturais (ajustado para IMC materno; modelo: comentários críticos, superenvolvimento emocional, enfrentamento desadaptativo)	- Mães de crianças obesas tiveram maior traço de ansiedade (z = -2,58; p = 0,01) e tendência à significância para estado de ansiedade (z = -1,85; p = 0,064). - Houve associação entre obesidade da criança e risco de depressão materna (z = 4,39; p = 0,036). - Na análise de trajetória, a depressão ou ansiedade materna não predisse diretamente o IMCz dos filhos (-0,13; p = 0,244). - Foram encontrados efeitos indiretos pela associação entre depressão ou ansiedade materna e superenvolvimento emocional (efeito indireto: 0,19; p = 0,006) e enfrentamento desadaptativo (efeito indireto: 0,09; p = 0,016).
de Castro et al., 2017 México n = 4.240 Transversal	Estudar a associação de sintomas depressivos maternos e desfechos de saúde e desenvolvimento infantil precoces em mães de baixo e alto nível socioeconômico.	Depressão materna (≥ 9) *Center for Epidemiologic Studies Depression Scale* (CES-D)	IMCz (> 2 DP) menores de 5 anos Wald para amostras complexas	- Não houve diferença significativa para excesso de peso dos filhos entre mães com e sem sintomas depressivos (10,5% *versus* 9,72%, respectivamente; p = 0,66).
Audelo et al., 2016 EUA (etnia mexicana) n = 332 Coorte	Investigar prospectivamente a associação entre sintomas depressivos em mulheres com filhos de idades entre 1 e 7 anos, e o sobrepeso e obesidade infantil aos sete anos em famílias latinas.	Depressão materna (moderada ≥ 16, severa ≥ 20) (1; 3,5 e 7 anos dos filhos) *Center for Epidemiologic Studies Depression Scale* (CES-D)	IMCp (sobrepeso ≥ 85, obesidade ≥ 95) 7 anos Regressão logística múltipla (número de anos nos EUA, peso pré-gestacional, tabagismo na gravidez, status de pobreza, densidade habitacional, apoio social, peso ao nascer, insegurança alimentar aos 7 anos)	- Filhos de mulheres com depressão recorrente (em 3 momentos) tiveram maior chance de apresentar sobrepeso ou obesidade aos 7 anos (OR = 2,4; IC95% 1,1–5,6). - Depressão materna em somente um ou dois pontos no tempo não mostrou nenhum efeito sobre o IMC da criança.
Brentani, 2016 Brasil n = 798 Transversal	Analisar a relação empírica entre depressão materna e desenvolvimento de crianças de 1 ano de idade usando dados de uma coorte.	Depressão materna (possível 10–13, provável ≥ 13) *Edinburgh Postnatal Depression Scale* (EPDS)	IMCz (> 2 DP) 12 meses Regressão linear multivariável (criança: idade, sexo, gemelar, PIG, prematuro; mãe: idade, escolaridade, estado civil, renda)	- Não foi encontrada associação entre depressão materna e obesidade das crianças (delta = 0,012; p > 0,05).
Morrissey & Dagher, 2014 EUA n = 6.500 Coorte	Estudar as associações precoces e contemporâneas entre sintomas depressivos maternos e IMC das crianças, obesidade e consumo alimentar.	Depressão materna (≥ 9) (9 meses; 2, 4 e 5,5 anos dos filhos) *Center for Epidemiologic Studies Depression Scale* (CES-D): 9 meses, 4 e 5,5 anos *Depression Scale of the Composite International Diagnostic Interview Short Form* (CIDI-SF): 2 anos	P/Cz (aos 9 meses)– (≥ p95) IMCz (2, 4 e 5,5 anos) – (≥ p95) Modelo linear de probabilidades (idade da criança, sexo, raça/etnia, seguro de saúde, peso pré-gestacional, horas de trabalho materno e paterno, estrutura familiar, situação de pobreza, área urbana ou rural; escolaridade dos pais)	- SDM foram associados a uma pequena diminuição na probabilidade de o filho ser obeso (0,8 pontos percentuais). - Maior duração dos sintomas depressivos maternos foi associada ao maior IMC (0,02 DP) entre as crianças com pais sem diploma universitário.
Wang et al., 2013 EUA n = 1.090 Coorte	Examinar a associação entre sintomas depressivos maternos durante a primeira infância dos filhos e, posteriormente, sobrepeso na infância.	Depressão materna (≥ 16) (1, 24 e 36 meses dos filhos) *Center for Epidemiologic Studies Depression Scale* (CES-D)	IMCp (≥ 85) 1ª, 3ª e 6ª séries Equação de estimativa generalizada (criança: peso ao nascer, raça, sexo; maternos: raça, idade, escolaridade, trabalho, estado civil, renda familiar, amamentação, tabagismo antes da gestação, suporte social)	- Filhos de mães deprimidas nos três momentos tiveram maior probabilidade de estar acima do peso do que os de mães nunca deprimidas: OR = 1,7; IC95% 1,01–2,87 no modelo ajustado para características da criança e OR = 2,13; IC95% 1,05–4,31 no modelo ajustado para características maternas.
Ramasubramanian, 2013 Reino Unido n = 10.465 Transversal	Examinar transversalmente a relação entre o sofrimento psicológico grave da mãe e a obesidade na primeira infância numa coorte de nascimentos.	Transtorno mental materno (≥ 13) (3 anos após o parto) *Kessler Psychological Distress Scale* (Kessler-6)	IMCp (≥ 95) 3 anos de idade Regressão logística multivariável (NSE, suporte familiar, escolaridade, trabalho materno, sobrepeso pré-gestacional, doença crônica materna)	- Sofrimento psíquico materno grave foi associado à obesidade infantil nas análises bruta (OR = 1,62; IC95% 1,11–2,37; p = 0,01) e ajustada (OR = 1,59; IC95% 1,08–2,34; p = 0,01).
Gross, 2013 EUA n = 401 Transversal	Caracterizar a relação entre sintomas depressivos maternos e o peso da criança, práticas de alimentação promotoras de obesidade e comportamentos relacionados à atividade física em famílias urbanas de baixa renda.	Depressão materna (leve 5–9, moderada a severa 10–29) *Patient Health Questionnaire-9* (PHQ-9)	IMCp (≥ 85) ≅ 5 anos Regressão logística multivariável (sexo, filho único; seguro de saúde, idade, escolaridade, raça, estado civil e situação de emprego maternos)	- Mães com sintomas depressivos moderados a graves tiveram maior probabilidade de ter filhos com excesso de peso do que mães sem sintomas (OR ajustada = 2,62; IC95% 1,02–6,70).
Gemmill, 2013 Austrália n = 159 Coorte	Investigar se os sintomas depressivos e ansiosos maternos pré-natais e/ou concomitantes eram preditivos do controle das práticas de alimentação infantil e examinar se o controle dessas práticas predizia o IMC infantil.	Depressão e ansiedade (NR) (gestação e 201507 anos) *Depression Anxiety Stress Scale* (DASS-21)	IMC (NR) 2–7 anos Regressão hierárquica múltipla (S1: renda familiar, escolaridade materna e idade; S2: número de crianças e sexo; S3: IMC na primeira infância, preocupação com o peso da criança, depressão, ansiedade e estresse)	- A depressão e ansiedade maternas não foram preditoras do IMC da criança (β = 0,24 e -0,14, respectivamente; p > 0,05), tampouco das práticas maternas de alimentação da criança (pressão para comer, restrição e monitoramento). Estas também não foram preditivas do IMC infantil.
McConley, 2011 EUA n = 4.601 Transversal	Esclarecer a relação entre a estrutura familiar, a depressão materna e o excesso de peso da criança, e se varia de acordo com a raça/etnia ou sexo da criança.	Depressão materna (NR) Subescala de depressão do *Brief Symptom Inventory* (BSI)	IMCp (sobrepeso 85–95, obesidade > 95) 5ª série Modelagem de equações estruturais (modelo: estrutura familiar, depressão materna, qualidade da parentalidade, comportamento sedentário, índice de alimentos saudáveis, atividades de lazer, IMC da criança)	- A associação entre SDM e IMC infantil foi mediada pela qualidade da parentalidade (EP = -0,09) e sua relação com a atividade de lazer (EP = -0,06) e comportamento sedentário das crianças (EP = 0,06) (p < 0,05).
Santos et al., 2010 Brasil n = 3.748 Coorte	Investigar a associação entre depressão materna pós-parto e crescimento da criança aos 4 anos.	Depressão materna (≥ 13) (1, 2 e 4 anos após o parto) *Edinburgh Postnatal Depression Scale* (EPDS)	P/Iz, (> 2 DP) 4 anos de idade Regressão logística múltipla (idade materna no parto, etnia, renda familiar, escolaridade, estado civil, tabagismo, IMC pré-gestacional, uso de serviços de saúde)	- O efeito dos SDM em um ou dois dos três acompanhamentos (OR = 1,0; IC95% 0,8–1,3) ou em todos os três (OR = 1,6; IC95% 1,0–2,5) não foi associado ao excesso de peso dos filhos.
Surkan et al., 2008 Brasil n = 589 Transversal	Avaliar se os sintomas depressivos maternos estão associados ao excesso de peso em crianças com idade entre 6 e 24 meses.	Depressão materna (≥ 16) (6 a 24 meses após parto) *Center for Epidemiologic Studies Depression Scale* (CES-D)	P/Cz (sobrepeso ≥ p85, obesidade > p95) 6 a 24 meses de idade Regressão logística múltipla (sexo, peso ao nascer, idade, duração da amamentação, escolaridade materna; escala de saneamento, NSE, número de filhos no domicílio; doença recente da criança e participação no Programa Saúde da Família)	- Sobrepeso e obesidade infantil foram maiores nos filhos de mães com sintomas depressivos elevados do que nos de mães assintomáticas (39,9% *versus* 28,5%; p= 0,004; e 20,9% *versus* 10,3%; p= 0,0005, respectivamente). - Filhos de mães com escores depressivos elevados tiveram chance quase duas vezes maior de sobrepeso (OR = 1,7; IC95% 1,4–2,2) e de obesidade (OR = 2,3; IC95% 1,6–3,3).
O’Brien, 2007 EUA n = 653 Coorte	Investigar correlatos ecológicos do desenvolvimento de excesso de peso em uma amostra de crianças acompanhadas por 12 anos.	Depressão materna (NR) (6, 15, 24, 36 e 54 meses após o parto e na 1ª, 3ª, 5ª e 6ª série dos filhos) *Center for Epidemiological Studies Depression Scale* (CES-D)	IMCp (≥ 85) 24, 36 e 54 meses; 1ª, 3ª, 5ª e 6ª séries Regressão logística múltipla (sexo e etnia da criança, escolaridade materna, renda familiar, estado civil, tempo que a criança vive em casa de pais separados)	- Depressão materna não diferiu entre os grupos de crescimento (nunca teve sobrepeso, sobrepeso pré-escolar, sobrepeso escolar e retorno ao peso normal [OR ≅ 1,0; p > 0,05]) e não foi preditora de pertencimento a um grupo específico.
Gibson, 2007 Austrália n = 329 Transversal	Investigar a relação entre peso da criança e uma ampla gama de fatores familiares e maternos.	Depressão e ansiedade (NR) *Depression Anxiety Stress Scale* (DASS-21)	IMCz (NR) 6–13 anos Regressão linear multivariável (IMC materno, escolaridade, renda, estrutura familiar, número de moradores em casa, sexo)	- Obesidade infantil não foi associada à depressão (β = 0,01 [IC95% -0,02; 0,04]) e ansiedade materna (β = 0,02 [IC95% -0,06; 0,06]).

IMC: índice de massa corporal; IMCz: escore-z de IMC; IMCp: percentil de IMC; NR: não reportado; RRR: razão de risco relativo; DP: desvio-padrão; PIG: pequeno para a idade gestacional; IC95%: intervalo de confiança de 95%; SDM: sintoma depressivo materno; RO: razão de odds; EP: estimativa padronizada; P/Cz: escore-z de peso para comprimento; P/Iz: escore-z de peso para idade; NSE: nível socioeconômico

### Achados Principais

Quanto aos resultados principais acerca da associação entre os sintomas maternos depressivos ou de ansiedade e o excesso de peso nos filhos, observou-se que, dos 16 estudos, oito encontraram uma associação positiva entre a exposição e desfecho de interesse, sendo um realizado no Brasil^16^ e sete em países de alta renda^9,12,21^. Desses últimos, a associação entre sintomas depressivos maternos e o IMC dos filhos teve variações de acordo com a cronicidade dos sintomas. Três desses estudos^21^, todos conduzidos nos Estados Unidos, encontraram associação com o excesso de peso dos filhos somente quando houve cronicidade dos sintomas depressivos maternos (medidas positivas para os sintomas em três acompanhamentos). No entanto, Morrissey et al.^22^ observaram essa associação somente em mães menos escolarizadas (sem formação universitária) e uma associação inversa com as mais escolarizadas.

O Quadro 1 apresenta uma síntese dos artigos incluídos nesta revisão sistemática.

## DISCUSSÃO

### Achados Principais

Esta revisão sistemática identificou 16 estudos que avaliaram a associação entre sintomas maternos de depressão e/ou ansiedade e excesso de peso dos filhos na infância e encontrou relação positiva em metade deles. Esse resultado parece ter sido influenciado pela qualidade da parentalidade das mães com sintomas depressivos ou ansiosos, ou seja, pela forma como funções e atividades são desenvolvidas por elas. Mães com sintomas depressivos podem ter menor qualidade parental, ou seja, menor habilidade para perceber e interpretar sinais e comunicações expressas no comportamento dos filhos, como menor cuidado com regras e rotinas das refeições e atenção à qualidade e quantidade dos alimentos consumidos^5,29^. O baixo controle da alimentação dos filhos pode levá-los a consumir alimentos menos saudáveis^22^ quando mais jovens e/ou a permitir escolhas alimentares não saudáveis quando mais velhos, fatores que corroboram para o maior ganho de peso^30,31^.

Além disso, outros comportamentos desenvolvidos e relacionados aos sintomas depressivos/ansiosos maternos podem explicar o excesso de peso da prole, entre eles: menor controle sobre o tempo de sono^25,29^, menor nível de atividade física^27^ e maior tempo de tela^12,20,27^. Nesse sentido, alguns dos estudos desta revisão encontraram associação positiva mediada por variáveis de estilo parental e comportamento da criança, como capacidade de resposta alimentar^26^, menor tempo de sono^25^, emoção expressa e enfrentamento desadaptativo^9^, restrição e monitoramento da alimentação dos filhos^25,26^, uso de alimentos como recompensa^25^ e menor atividade física das crianças^12,25,27^.

Essas práticas, que envolvem participação direta e ativa da mãe, podem ser afetadas negativamente pela depressão devido a sintomas como perda de interesse, baixa energia e fadiga^25,32^. Mães deprimidas geralmente são menos responsivas ao filho e escolhem estratégias de enfrentamento que exigem menor esforço cognitivo^3,32^. A parentalidade permissiva, que ocorre quando a mãe coloca poucas exigências e deixa de estabelecer limites no comportamento da criança, tem sido positivamente associada à obesidade infantil^3,12,32^. Filhos de mães deprimidas apresentam maiores taxas de insucesso em programas de intervenção no peso^33^, sinalizando que o rastreamento e o tratamento da depressão materna podem ajudar a melhorar os resultados relativos ao estado nutricional das crianças.

Do total de artigos incluídos nesta revisão, cinco foram realizados em países de média renda e apenas um deles, com delineamento transversal, encontrou associação entre sintomas depressivos maternos e excesso de peso dos filhos (entre 6 e 24 meses de idade)^16^. Uma vez que famílias de baixa renda têm um maior risco tanto de obesidade^1^ quanto de depressão materna^3^, uma possível explicação para a não associação pode ser a interação entre fatores socioeconômicos e socioculturais na determinação do efeito da saúde mental materna sobre a nutrição infantil^13,15,30^. Isto é, famílias com menor nível econômico, em geral, possuem menores níveis de conhecimento e de poder de compra, que afetariam a nutrição adequada do filho^34^. Apesar de fugir ao escopo desta revisão, é importante ressaltar que alguns estudos encontraram ligação entre sintomas depressivos maternos e baixo peso da criança^15^ ou menor peso do que filhos de mães sem sintomas depressivos^22,31^. No entanto, a relação parece ocorrer principalmente em países de menor renda ou em populações socioeconomicamente carentes residentes em países de maior renda.

Os resultados divergentes entre os estudos desta revisão também podem ser reflexo das diferenças metodológicas. Um dos fatores metodológicos que variou entre eles foi o momento de avaliação da exposição da criança aos transtornos mentais maternos, variando de abaixo de 1 ano a 12 anos. Essa variação pode ter interferido nos resultados de duas formas: (1) talvez exista uma janela de idade sensível à exposição ao transtorno para posterior desenvolvimento da obesidade, e estudos que não contemplaram esse período sensível podem não ter encontrado associação; (2) talvez essa associação seja afetada pela cronicidade da exposição, e o efeito da exposição aos transtornos maternos se manifesta quando há uma longa exposição. Corroborando com a limitação para a comparação entre estudos, o período em que os sintomas maternos de depressão e ansiedade foram medidos não apresentaram um padrão consistente com a medida de IMC dos filhos. Não está claro qual seria o momento mais específico para mensurar os sintomas depressivos maternos para melhor predizer o risco de excesso de peso dos filhos^11,35^.

### Limitações dos Estudos

Dentro das limitações, sete autores pontuaram as perdas ocorridas nos estudos com delineamento longitudinal, as quais muitas vezes não puderam ser classificadas^12,15,16,21^. A possibilidade de viés de não resposta também foi considerada^12,22,27^. Esses vieses podem afetar o resultado dos estudos, uma vez que que mães com sintomas depressivos graves são mais susceptíveis a não participar ou abandonar o estudo, diminuindo a magnitude ou mesmo suprimindo a associação. Outra limitação apontada refere-se à atribuição de causalidade, inerente aos estudos com delineamento transversal, quando as medidas dos sintomas depressivos ocorreram concomitantemente à realização da antropometria da criança^8,16,24,25^. Portanto, assim como o transtorno mental materno pode afetar o peso da criança, mães de crianças com excesso de peso podem ter sintomas depressivos em consequência dessa condição, inviabilizando a inferência causal da relação. Apesar dessas limitações, os resultados dos estudos não foram invalidados, pois os autores utilizaram técnicas para minimizar esses problemas, como imputação de dados faltantes e análises estatísticas adequadas, considerando e discutindo as limitações em seus trabalhos.

O fato de as informações terem sido relatadas pelas mães pode ter prejudicado a validade das respostas, visto que as com sintomas depressivos são menos propensas a monitorar as atividades e comportamentos das crianças. Não está claro, no entanto, se os sintomas depressivos maternos levariam ao excesso ou ao sub-relato dessas rotinas e comportamentos. O foco dos estudos na depressão materna também foi apontado como uma possível fonte de erro de informação, correlacionado ao viés de desejabilidade social^28^.

Alguns autores apontaram a falta de dados sobre IMC materno e a ausência ou baixa qualidade das informações sobre a alimentação da criança como limitações^12,16^. O primeiro pode confundir a associação ou estar no caminho causal entre o transtorno mental materno e o peso da criança, enquanto o segundo pode ser um mediador da associação. Limitações nas medidas de consumo alimentar também foram consideradas, em alguns casos explicando a menor magnitude^16,22^ ou mesmo a ausência de associação^18,20,27^. Algumas razões discutidas para a não associação incluíram amostra de conveniência^18^, perdas de acompanhamento^18,20,28^ e possível viés de relato dos sintomas depressivos^18,28^, assim como a possibilidade de confusão residual^15^. Conforme discutido, esses fatores podem ter inviabilizado o resultado positivo da associação nestes estudos.

Foram encontrados apenas três estudos que abordaram os sintomas de ansiedade maternos, juntamente com os depressivos, o que poderia ajudar na melhor compreensão dessa relação, visto que quadros ansiosos podem evoluir para depressão ou coexistir com ela. Apenas em um desses trabalhos a associação foi significativa^8^, porém ansiedade e depressão foram agrupadas em um construto único definido como psicopatologia materna, impossibilitando atribuir a associação a cada transtorno individualmente.

### Pontos Fortes dos Estudos

Entre os pontos fortes da metodologia dos estudos, destaca-se o grande número de indivíduos pesquisados, alguns deles com representatividade nacional^12,16,24,28^. Como ponto positivo, a maioria realizou a coleta de dados antropométricos por entrevistadores treinados e padronizados. Os estudos longitudinais permitem estabelecer uma relação temporal entre os sintomas de depressão maternos e a obesidade infantil, além de a medida dessa sintomatologia ter sido realizada em vários acompanhamentos, sendo possível distinguir sintomas crônicos de sintomas episódicos.

Metodologicamente, o presente estudo buscou pesquisas que coletassem informações sobre os transtornos mentais maternos durante a infância dos filhos, visto que a primeira infância (dois a cinco anos de idade) foi identificada como um período crítico, no qual são estabelecidos os comportamentos (atividade física e dieta) que influenciam a obesidade^36^. Esta revisão buscou discutir os achados considerando que as pesquisas foram conduzidas com populações de locais distintos, fator que pode ter influenciado na presença ou ausência da associação e que, consequentemente, precisa ser ponderado na comparação dos resultados. A heterogeneidade entre os estudos em relação à mensuração e categorização da depressão materna e idade da criança na exposição e no desfecho inviabilizou a demonstração de uma estimativa combinada dos efeitos em metanálise, o que poderia melhor explicar os resultados. Em relação à qualidade dos trabalhos selecionados, a maioria apresentou um bom desempenho na avaliação realizada, sendo que apenas dois^9,19^ obtiveram pontuação abaixo de 10 pontos, do total de 15.

No mesmo sentido dos resultados descritos, revisões anteriores^10,11,35^ encontraram uma relação positiva entre depressão^11,35^ e outras psicopatologias^11^ maternas e obesidade nos filhos, com resultados divergindo de acordo com a medida do transtorno materno (pré ou pós-natal, de forma isolada ou longitudinal) e com as diferentes faixas etárias das crianças (pré-escolares, infância e adolescência). Os estudos foram realizados nos anos de 2012, 2014 e 2015 e incluíram, respectivamente, 5, 9 e 20 trabalhos. Embora apresentem algumas diferenças em relação aos critérios de inclusão, evidenciam o crescente interesse sobre o tema nos últimos anos.

### Considerações Finais

Apesar da associação positiva em grande parte dos estudos, há que se considerar as discordâncias entre os resultados observados, indicando que a saúde mental materna contribui na promoção da obesidade infantil, porém atua em conjunto com outros fatores envolvidos em sua etiologia. Para um melhor entendimento desse fenômeno, é importante que fatores de risco paternos e familiares, como a saúde mental, também sejam investigados.

Algumas medidas para o enfrentamento do problema exposto são recomendadas, como o rastreamento de transtornos mentais maternos desde o pré-natal e, após o parto, nas consultas de rotina da criança. Em países de maior vulnerabilidade social que contem com equipes de saúde domiciliar, o diagnóstico e tratamento desses transtornos pode ser mais facilmente oportunizado por meio de treinamento dos profissionais de saúde. Intervenções de saúde comunitária em países de baixa e média renda que incluíram um componente de saúde mental materna (visita domiciliar, escuta empática e técnicas cognitivo-comportamentais) demonstraram sucesso no declínio dos sintomas depressivos nas mulheres do grupo intervenção^37^. Com base nesta revisão, conclui-se que há evidências de uma relação entre os sintomas de depressão crônica materna e o excesso de peso dos filhos, mediada por comportamentos da mãe e da criança. Portanto, é fundamental aprofundar o conhecimento acerca dos mecanismos que influenciam nessa associação para que políticas de cuidado à saúde mental das mulheres possam ser desenvolvidas, impactando indiretamente no estado nutricional das crianças.
